# Placenta Accreta Spectrum Disorders – The Impact of the Creation of a Multidisciplinary Team on Maternal Outcomes in Portugal

**DOI:** 10.1055/s-0043-1772482

**Published:** 2023-12-23

**Authors:** Beatriz Teixeira, Pedro Viana Pinto, Rodrigo Realista, Manuela Silva, Antónia Costa, Ana Paula Machado, Marina Moucho

**Affiliations:** 1 Department of Obstetrics and Gynecology, Centro Hospitalar Universitário de São João, Porto, Portugal; 2 Department of Anatomy, Faculdade de Medicina, Universidade do Porto, Porto, Portugal; 3 Department of Obstetrics and Gynecology, Faculdade de Medicina, Universidade do Porto, Porto, Portugal

**Keywords:** placenta accreta spectrum disorders, multidisciplinary team, maternal morbidity, distúrbios do espectro da placenta acreta, equipe multidisciplinar, morbidade materna

## Abstract

**Objective** To describe a cohort of placenta accreta spectrum (PAS) cases from a tertiary care institution and compare the maternal outcomes before and after the creation of a multidisciplinary team (MDT).

**Methods** Retrospective study using hospital databases. Identification of PAS cases with pathological confirmation between 2010 and 2021. Division in two groups: standard care (SC) group – 2010–2014; and MDT group – 2015–2021. Descriptive analysis of their characteristics and maternal outcomes.

**Results** During the study period, there were 53 cases of PAS (24 - SC group; 29 - MDT group). Standard care group: 1 placenta increta and 3 percreta; 12.5% (3/24) had antenatal suspicion; 4 cases had a peripartum hysterectomy – one planned due to antenatal suspicion of PAS; 3 due to postpartum hemorrhage. Mean estimated blood loss (EBL) was 2,469 mL; transfusion of packed red blood cells (PRBC) in 25% (6/24) - median 7.5 units. Multidisciplinary team group: 4 cases of placenta increta and 3 percreta. The rate of antenatal suspicion was 24.1% (7/29); 9 hysterectomies were performed, 7 planned due to antenatal suspicion of PAS, 1 after intrapartum diagnosis of PAS and 1 after uterine rupture following a second trimester termination of pregnancy. The mean EBL was 1,250 mL, with transfusion of PRBC in 37.9% (11/29) - median 2 units.

**Conclusion** After the creation of the MDT, there was a reduction in the mean EBL and in the median number of PRBC units transfused, despite the higher number of invasive PAS disorders.

## Resumo

**Objetivo** Descrever uma coorte de casos do espectro do acretismo placentário (PAS) de uma instituição terciária e comparar os resultados maternos antes e depois da criação de uma equipa multidisciplinar (MDT).

**Métodos** Estudo retrospectivo utilizando bancos de dados hospitalares. Identificação de casos de PAS com confirmação patológica entre 2010 e 2021. Divisão em dois grupos: grupo *Standard Care* (SC) – 2010–2014; e grupo MDT – 2015–2021. Análise descritiva de suas características e desfechos maternos.

**Resultados** Durante o período do estudo, houve 53 casos de PAS (24 - grupo SC; 29 - grupo MDT). Grupo Standard Care: 1 placenta increta e 3 percretas; 12,5% (3/24) tiveram suspeita anteparto; 4 casos tiveram histerectomia periparto – uma eletiva devido à suspeita anteparto de PAS; 3 devido a hemorragia pós-parto. A média de perda hemática estimada (EBL) foi de 2.469 mL; transfusão de concentrado eritrocitário (PRBC) em 25% (6/24) - mediana 7,5 unidades. Equipa multidisciplinar: 4 casos de placenta increta e 3 percretas. A taxa de suspeita anteparto foi de 24,1% (7/29); foram realizadas 9 histerectomias, 7 eletivas por suspeita anteparto de PAS, 1 após diagnóstico intraparto de PAS e 1 após rotura uterina após interrupção da gravidez no segundo trimestre. A EBL média foi de 1.250 mL, com transfusão de PRBC em 37,9% (11/29) - mediana de 2 unidades.

**Conclusão** Após a criação da MDT, houve redução na média de EBL e na mediana do número de unidades de PRBC transfundidas, apesar do maior número de PAS invasivos.

## Introduction

Placenta accreta spectrum (PAS) disorders are an abnormality in placentation in which the chorionic villi adhere or invade the myometrium to a variable extent. 1 This complex clinical entity is associated with important morbidity, not only due to heavy bleeding but also to invasion of the surrounding pelvic organs by placental tissue. In a recent meta-analysis revising the main outcomes of PAS, 46.9% of the cases were complicated by hemorrhage, requiring transfusion. 2

Although, historically, PAS is a rare complication of pregnancy, its incidence has been rising in the last decades, with recent reported occurrences in 1:533 to 1:730 deliveries. 3 4 This is often attributed to the increasing cesarean rates, as there is an association between the number of prior cesarean deliveries and PAS, particularly in the presence of placenta previa. 5 Other risk factors include a previous pregnancy with PAS, in vitro fertilization, and antecedents of manual removal of the placenta or uterine surgeries, such as curettage, endometrial ablation, and myomectomy. 6 7 8

The most widely accepted treatment for PAS is cesarean hysterectomy with the placenta left in situ. 9 10 11 There is also a consensus in the recommendation that PAS should be treated by a multidisciplinary team (MDT) with expertise in complex pelvic surgery. 9 10 11 The MDT approach has been associated with better maternal outcomes, such as a reduction in estimated blood loss with lower transfusion requirements and a lower likelihood of reoperation within a week of delivery. 12 13 14 15 16 17 18

The aim of the present study was to describe the cohort of PAS cases from a tertiary care institution and to compare the maternal outcomes before and after the creation of a dedicated MDT.

## Methods

This is a retrospective cohort study including all the cases of PAS confirmed histologically that were treated in a tertiary teaching hospital in Porto, between 2010 and 2021. The diagnosis of PAS was made by identification of myometrial tissue adjacent to the chorionic villi (with immunohistochemical confirmation) with focal interruption of the decidua basalis, according to the classification by Hecht et al. 19 The cases were grouped according to the FIGO classification for PAS in placenta accreta (grade 1), increta (grade 2), and percreta (grades 3a–3c), 1 based on intraoperative findings and the pathology report. The cases of PAS were identified by the search of various key terms (low-lying placenta; manual removal of the placenta; peripartum hysterectomy; postpartum hemorrhage) in hospital-based electronic databases Obscare – Virtual Care (Porto, Portugal) and S Clínico – SPMS (Lisbon, Portugal).

After review of the medical records, data was collected regarding baseline characteristics (age, body mass index, parity and mode of conception); risk factors for PAS disorders (placenta previa, prior caesarean section, and number and history of other uterine surgeries); timing of diagnosis; antepartum and intrapartum management; gestational age at delivery; neonatal outcomes (birthweight, neonatal intensive care unit [NICU] admission, and Apgar scores); immediate maternal morbidity; reoperations (early or delayed, defined as urgent surgical procedures performed during the first 7 days after birth/spontaneous abortion or after 7 days, respectively). Early maternal morbidity was defined as the occurrence of one or more of the following: maternal admission to the intensive care unit (ICU), transfusion of packed red blood cells (PRBCs), coagulopathy, ureteral or bladder wall injury, or early reoperation (up to 7 days after the surgery). Late maternal morbidity was defined as the occurrence of one or more of the following: hospital re-admission within 6 weeks or delayed reoperation (performed between 7 days to 6 weeks after the surgery).

The standard care (SC) group was composed by the cases occurring before 2015 (prior to the creation of the MDT) and were managed on a case-by-case basis, as defined by the medical team. This approach could include formal or informal consultations with other specialties. In this group, the management of patients with antenatal suspicion of PAS ranged from planned peripartum hysterectomy with the placenta left in situ (with the possibility of placement of balloon catheters on the common iliac arteries as decided by the surgeons) to the performance of cesarean sections with an attempt to remove the placenta.

The MDT group included the cases treated in our institution after the creation of the MDT in 2015. Our team includes members from the following areas: maternal-fetal medicine, gynecological oncology, obstetrical ultrasound, interventional radiology, and urology; consultations were also made with the blood bank, anesthesiology, intensive care unit and neonatal intensive care unit. In cases with suspicion of deeper invasion, ultrasound evaluation of the placenta by a dedicated sonographer and magnetic resonance imaging of the placenta are performed. Whenever possible, the delivery is planned to the late preterm period (35–37 weeks of gestation). The woman is electively admitted for preoperative optimization and planning: a cycle of corticosteroids for fetal lung maturity is performed according to the gestational age. Adequate blood products should be available at the time of delivery for scheduled PAS and are prepared in advance.

On the day of the surgery for cases of a suspicion of higher degree invasion, in the same operative room, the patient is submitted to a cystoscopy with evaluation of bladder involvement and placement of ureteric stents; catheters on the common iliac arteries are also placed (inflated intraoperatively, if necessary). When a cesarean hysterectomy is performed, it is initiated under locoregional anesthesia and converted to general anesthesia after fetal extraction. A vertical midline skin incision is performed, and the uterus is exteriorized. The hysterotomy is made above the superior border of the placenta, usually in a fundal location. After fetal extraction, the placenta is left in situ with no attempts to remove it and no uterotonics are used. After closure of the uterine incision, either a simple or a modified radical hysterectomy is performed (depending on the degree of placental invasion), with a bilateral opportunistic salpingectomy, in accordance with recent recommendations for reduction of the risk of ovarian cancer. 20

For the study, ‘elective’ deliveries were classified as deliveries performed non-urgently in prearranged designated theaters at least 24 hours in advance. ‘Emergency’ deliveries were deliveries performed expeditiously due to concerns for either maternal or fetal wellbeing. Reasons for emergency deliveries are discussed further.

The two groups were characterized using descriptive statistics. Due to the design of the study and the number of cases, no other statistical tests were performed. Approval was obtained from the ethics committee of Centro Hospitalar e Universitário de São João.

## Results

During the study period, there were 29,517 deliveries, with 53 cases of histologically confirmed PAS disorders identified, making an incidence of 1.8/1,000. Of these, 24 occurred before 2015 and were part of the SC group; 29 were in the MDT group (2015–2021). The maternal baseline characteristics are presented in Table 1 . In the SC group, half of the patients (12/24) were primiparous, and 5 had previous cesarean deliveries (20.8%). Regarding other risk factors for PAS, one patient had been submitted to previous hysteroscopic procedures (three ressectoscopic myomectomies), and there were 6 cases of placenta previa (25%). As for the MDT group, the rate of primiparous patients was 24.1% (7/29) and in more than half of the cases (51.7% - 15/29), there was a history of previous cesarean deliveries. Most cases corresponded to placenta accreta (FIGO grade 1), as expected (SC group – 20/24 [83.3%]; MDT group – 22/29 [75.9%]).

**Table 1:** Maternal characteristics

Characteristics	SC group ( *n* = 24)	MDT group ( *n* = 29)	Total ( *n* = 53)
Maternal age in years, median (range)	33.5 (23–44)	37 (25–43)	36 (23–44)
Pregestational BMI (kg/m ^2^ ), median (range)	25.4 (18.5–47.5)	23.7 (17.3–30.4)	24.3 (17.3–47.5)
Assisted reproductive techniques – n	3	1	4
Primiparous – n (%)	12 (50)	7 (24.1)	19 (35.8)
Gestational age at delivery, median (range)	38 (32–41)	38 (27–41)	38 (27–41)
Previous cesarean delivery – n (%)	5 (20.8)	15 (51.7)	20 (37.7)
Number of previous cesarean deliveries – n (%)			
0	19 (79.2)	14 (48.3)	33 (62.3)
1	4 (16.7)	12 (41.3)	16 (30.2)
2 or more	1 (4.2)	3 (10.3)	4 (6.9)
Other uterine surgical procedures - n	1	1	2
Placenta previa – n (%)	6 (25)	7 (24.1)	13 (24.5)
Placenta previa and previous cesarean deliveries – n (%)	1 (4.2)	6 (20.7)	7 (13.2)
No identifiable risk factors for PAS	14 (58.3)	10 (34.5)	24 (45.3)

Abbreviations: BMI, body mass index; MDT, multidisciplinary team; n, number; SC, standard care.

The antenatal and intrapartum characteristics in both groups are summarized in Table 2 . There was an antenatal suspicion of PAS in 3 cases of the SC group (1 case FIGO grade 1, 1 FIGO 2, and 1 FIGO 3) and 7 cases of the MDT group (corresponding to the 7 cases of abnormally invasive placenta). Of the SC cases with antenatal suspicion of PAS, in one of them a planned peripartum hysterectomy was performed with the placenta left in situ. In the other two, the delivery was made by a scheduled cesarean section, with attempts to remove the placenta that culminated in a hysterectomy to control the postpartum hemorrhage (PPH) in one instance. As for the 7 cases of the MDT group that were identified antenatally, all were submitted to a planned peripartum hysterectomy by the MDT, following the protocol described. Balloon catheters in the common iliac arteries were placed in 1 patient in the SC group (inflated in that case) and in 7 cases in the MDT group (inflated in 3 cases).

**Table 2:** Antenatal and intrapartum characteristics

	SC group ( *n* = 24)	MDT group ( *n* = 29)	Total ( *n* = 53)
Type of PAS – n (%)			
Accreta (FIGO Grade 1)	20 (83.3)	22 (75.9)	42 (79.2)
Increta (FIGO Grade 2)	1 (4.2)	4 (13.8)	5 (9.4)
Percreta (FIGO Grade 3)	3 (12.5)	3 (10.3)	6 (11.3)
Grade 3a	2 (8.4)	1 (3.4)	3 (5.7)
Grade 3b	1 (4.2)	1 (3.4)	2 (3.8)
Grade 3c	0 (0)	1 (3.4)	1 (1.9)
Antenatal suspicion of PAS – n (%)	3 (12.5)	7 (24.1)	10 (18.9)
Intrapartum suspicion of PAS – n (%)	13 (54.2)	15 (51.7)	28 (52.8)
Gestational age at delivery, median (range)	38 (32–41)	38 (27–41)	38 (27–41)
Mode of delivery – n (%)			
Cesarean section	13 (54.2)	18 (62.1)	31 (58.5)
Vaginal delivery	11 (45.8)	8 (27.6)	19 (36.5)
Birthweight in grams (mean)	2,926	2,732	2,772
Hysterectomy – n	4	9	13
Planned	1	7	8
Urgent (in the setting of PPH)	3	1 a	4
After intrapartum diagnosis of PAS disorder	0	1	1
Placement of catheters on common iliac arteries – n	1	7	8
Inflated – n	1	3	4

Abbreviations: FIGO, International Federation of Gynecology and Obstetrics; MDT, multidisciplinary team; n, number; PAS, placenta accreta spectrum; PPH, postpartum hemorrhage; SC, standard care.

a Hysterectomy performed after uterine rupture in the context of a second-trimester termination of pregnancy.

Table 3 summarizes the maternal and neonatal morbidity associated with PAS in this series. In the SC group, the mean estimated blood loss (EBL) was 2,469 mL, with a need for transfusion of PRBCs in 25% (6/24) and a median of 7.5 units of PRBCs transfused. In the MDT group, the EBL was 1,250 mL, and there was a need for transfusion of PRBCs in 11 cases (37.9% - median of 2 units). Prior to 2015, a total of 6 cases fulfilled the criteria previously defined for early maternal morbidity; after 2015, there were 11 cases of early maternal morbidity.

**Table 3:** Maternal and neonatal morbidity

	SC group ( *n* = 24)	MDT group ( *n* = 29)	Total ( *n* = 53)
Estimated blood loss in mL (mean, range)	2,469 (450–8,200)	1,250 (450–4,500)	1,807 (450–8,200)
Early maternal morbidity - n	6	11	17
ICU admission	4	2	6
Need for PRBC transfusion – n (%)	6 (25%)	11 (37.9%)	17 (32.1%)
Number of PRBC units (median, range)	7.5 (2–12)	2 (1–7)	5,5 (1–12)
Coagulopathy	2	0	2
Bladder injury	2	1	3
Ureteral injury	1	0	1
Early re-operation	2	0	2
Late maternal morbidity - n	2	1	3
Hospital re-admission	2	1	3
Delayed re-operation	1	1	2
Neonatal morbidity – n			
NICU admission	5	7	12
5 minute Apgar score < 7	2	0	2

Abbreviations: ICU, intensive care unit; MDT, multidisciplinary team; n, number; NICU, neonatal intensive care unit; PRBC, packed red blood cells; SC, standard care.

As for the late maternal morbidity, there were 2 cases of hospital readmission in the SC group – one of them motivated by a pyelonephritis in a patient with ureteric stents and the other by a late postpartum hemorrhage that required two suction curettages for complete removal of placental fragments. The MDT group had one readmission in a patient that had a second trimester termination for a fetus with trisomy 21 and presented with a pelvic abscess that motivated surgical treatment culminating in a hysterectomy.

Regarding the neonatal outcomes, there were 5 admissions to the NICU in the SC group and 7 in the MDT group. There was a fetal death in the first group, subsequent to the rupture of an undiagnosed vasa previa at 32 weeks of gestation. In the latter group, in addition to the termination of pregnancy described above, there was one uterine rupture at 20 weeks in a woman with a history of a previous cesarean delivery and a hysteroscopic septostomy.

Looking only at the cases of deeper placental invasion (FIGO grades 2 and 3), there were 4 cases in the SC group and 7 cases in the MDT; half of them were antenatally suspected in the SC group and all of them were suspected in the MDT group; the mean EBL was 5,300 mL in the SC group (in 3 hysterectomy was not planned antenatally), with a universal need of transfusion of PRBCs (median of 7.5 units). In the MDT group, all cases were managed with planned hysterectomy; the mean EBL was 1,614 mL, with transfusion of PRBCs required in 5 of the 7 cases (median of 1 unit).

## Discussion

The present study describes a cohort of PAS of a tertiary hospital, looking at two different groups in different periods of time: a group of cases when there was no standardized protocol regarding the management of these situations and the most recent group, after the creation of a MDT. After 2015, there were more cases with deeper placental invasion (4 of placenta increta – FIGO 2 - and 3 of percreta – FIGO 3), a result of referral from other centers to the newly established team. This factor, combined with a greater percentage of women with a previous cesarean delivery and placenta previa, probably explains the higher rate of antenatal suspicion of PAS in the MDT group; in this group, all the cases with higher degrees of invasion were identified antenatally (7/7). Despite having more cases of PAS FIGO 2 and 3, the mean EBL and the median number of PRBC units transfused were lower (1,250 versus 2,469 mL; 2 versus 7.5 units). The difference is even more pronounced in cases of PAS FIGO 2 and 3: mean EBL of 5,300 mL and transfusion of a median of 7.5 units of PRBC units in the SC group versus 1,614 mL and 1 unit, respectively, in the MDT group. Our results are in line with the evidence of lower maternal morbidity in cases of PAS managed by a MDT. 12 13 14 15 16 17 18 Antepartum suspicion of PAS is paramount to the management of this condition by MDT, leading to the improvement of maternal outcomes. 21 22 The combination of anterior placenta previa and a prior cesarean delivery should alert for the high risk of PAS, as highlighted by several guidelines on this subject. 23

Placenta accreta (FIGO grade 1), the more benign of PAS disorders, accounts for most of the cases in our study. When compared with invasive PAS (FIGO grades 2 and 3), this clinical entity is associated not only with a lower rate of antenatal diagnosis but also with less severe maternal morbidity. 24 This is congruent with our findings, as most of these cases were not identified antenatally, and there was even a subset that had no intrapartum suspicion, with the diagnosis of placenta accreta made incidentally in the pathology exam. Most of these cases could be classified as basal plate myometrial fibers, according to Hecht et al. 19

The absence of antenatal suspicion of PAS or placenta previa in most cases explains the relatively high gestational age at delivery (38 weeks), even in the MDT group. This, as well as the fact that an important subset of patients had no identifiable risk factors for PAS (i.e., previous cesarean sections, previous uterine surgeries, or even medically assisted pregnancies), highlights the importance of clinical awareness for this entity and the implementation of best practice protocols in the event of intrapartum diagnosed PAS. In the absence of the MDT, every obstetrician should be familiarized with the basic management of PAS to ensure the best possible maternal outcomes, remembering that the cases are not always antenatally suspected and that even FIGO 1 cases can be associated with important morbidity. In cases of intrapartum suspicion, it is important to be aware of different approaches, from leaving the placenta in situ (at least the attached fragments) to hysterectomy (immediate or delayed, according to the emergency of the situation and the availability of the MDT).

As the incidence of PAS rises, and with the growing experience of the MDTs, the management of this condition is constantly evolving. 25 An example of this is the recently described Soleymani-Alazzam-Collins (SAC) technique regarding the surgical treatment of severe PAS. 26 Our center has also adopted a similar approach over the years, getting closer to the SAC technique in some points, such as the transversal skin incision, the identification and slinging of the ureters without catheter placement, and the identification of the common and internal iliac arteries (with the objective of reducing the need for balloons in the common iliac arteries). There is conflicting evidence in the literature regarding the need for placement of vascular balloons. 27 28 While some authors regard them as very useful, others believe that with a surgically experienced team with there may be no need for them. 29

Our study has some limitations. First, its retrospective nature and the small sample size did not allow us to perform statistical tests comparing both groups. Another potential weakness in our study is the subjectivity of EBL in our primary outcome, which may have biased the study and the poor data obtained for the SC group. In fact, the percentage of PRCB transfusion is higher in the MDT group despite a lower mean EBL, which may be explained by a more aggressive approach toward anemia in the contemporary practice as well as by the difficulty of obtaining good data in the SC group. Also, some of the less severe cases had no EBL recorded. With the introduction of our multidisciplinary approach, the rigor of blood loss estimation was significantly increased. If anything, patients in the MDT group were more likely to have their blood loss overestimated and to receive more blood than the patients managed without this approach. Lastly, although we consider the histopathological definition of PAS in our study to be a strength, allowing for a more objective and standardized classification, 1 19 it also raises some limitations. The pathological examination of delivered placentas is more challenging when compared with hysterectomy samples. There is still some uncertainty in the literature regarding the clinical significance of basal plate myometrial fibers, as this entity may include cases of placenta accreta, with increased maternal morbidity, but may also be an incidental finding. 19 There is also the possibility that our study design might miss cases of PAS that had no antenatal suspicion and no pathological examination of the placenta. However, considering the limitations mentioned above, we believe that these cases probably corresponded to situations with low morbidity, and, therefore, their clinical significance is dubious.

## Conclusion

In conclusion, our study describes the maternal outcomes of PAS cases before and after the creation of a specialized MDT. Despite the higher number of invasive PAS disorders, there was a reduction in the mean EBL, highlighting the importance of a MDT for diagnosis and treatment of PAS cases.
